# Knowledge and Awareness of MTCT and PMTCT Post-Natal Follow-up Services Among HIV Infected Mothers in the Mankweng Region, South Africa

**DOI:** 10.2174/1874613601711010036

**Published:** 2017-06-30

**Authors:** Refilwe Ramoshaba, Sello Levy Sithole

**Affiliations:** 1 Department of Sociology and Anthropology, Faculty of Humanities, School of Social Sciences, University of Limpopo, Sovenga, South Africa; 2 Department of Social work, School of Social Sciences, Faculty of Humanities, University of Limpopo, Sovenga, South Africa

**Keywords:** Awareness, Follow-up, Knowledge, Mother-to-Child Transmission, PMTCT

## Abstract

**Background:**

The pandemic of Human Immunodeficiency Virus (HIV) is the most severe health challenge affecting children across the world. It is estimated that more than 90% of all HIV infections in children result from Mother-to-Child Transmission (MTCT). Poor knowledge and awareness of MTCT and Prevention of Mother-to-Child Transmission (PMTCT) among HIV positive mothers and their babies is a major setback to the success of the PMTCT programmes.

**Methods:**

A qualitative approach and a cross-sectional design were applied in this study. The sample size of the study was 26 participants. Purposive sampling was used to select HIV infected mothers enrolled for PMTCT follow-up services and health care providers responsible for the implementation of the PMTCT programmes. In-depth interviews were conducted with fifteen HIV infected mothers at two health facilities. Two Focus Group Discussions (FGDs) were conducted with eleven health workers at the two health facilities. Focus groups comprised of six participants from Mankweng Clinic and five participants from Mankweng Gateway Clinic.

**Results:**

The findings from the study reveal that the majority of the respondents were aware of MTCT, but lacked knowledge and understanding about how a mother can transmit HIV to her child during pregnancy, labour and breastfeeding. The majority of the participants did not understand the risk of MTCT after birth and failed to mention breastfeeding as a mode of transmission. However, most of the participants were aware that MTCT can be prevented. The respondents were aware of the importance of treatment adherence as a prevention measure to avoid MTCT.

**Conclusion:**

Based on these findings, a number of recommendations were made. The first is that educational and awareness programmes need to be developed or strengthened on health risks. Mass campaign media should provide information on the importance of PMTCT activities through the use of community radio stations, Television, newsletters, bill boards etc. People need to know more about PMTCT activities, health personnel need continuous training to provide clear information on PMTCT activities.

## INTRODUCTION

The pandemic of HIV is the most severe health challenge affecting children across the world [1-3]. The devastating source of HIV infection in children occurs through MTCT. Mother-to-Child Transmission results in more than 90% of all HIV infection in children around the world [4, 5]. It is estimated that 3.2 million children were living with HIV worldwide in 2013, and 360 000 of these children were from South Africa [5]. Global scale up of interventions to prevent MTCT has reduced new infections by 70% between the year 2009 and 2015. In 2015, approximately 1.8 million children were living with HIV worldwide [6]. Despite the global scale up of interventions, paediatric infections of HIV still remain high.

The implementation of PMTCT programmes has become one of the most important areas of intervention that prevents the spread of HIV [1, 2]. PMTCT interventions help prevent HIV infections from a mother living with HIV to her child during pregnancy, labour or delivery and breastfeeding [5]. In South Africa, the Department of Health introduced the PMTCT programmes in 2001, where a single dose of nevirapine was administered as an antiretroviral (ARV) prophylaxis for both infants and their mothers. In 2008, the procedure was updated to a dual therapy protocol that added Zidovudine (AZT) to the treatment regimen [1].

The aim of PMTCT programmes is to reduce the spread of HIV from mothers to their babies, but the identification and elimination of barriers that influence the use of PMTCT postnatal follow-up service has proven to be a challenge for programme planners and healthcare workers in implementing successful PMTCT programmes [1, 7]. Lack of awareness and knowledge impact negatively on the up-take of PMTCT postnatal follow-up services [8]. PMTCT programmes around the world are hugely affected by the level of awareness and knowledge of HIV and Acquired Immune Deficiency Syndrome (AIDS) as well as MTCT, which consequently increases HIV prevalence among children [9]. In the PMTCT programme, postnatal follow-up services are provided for mothers and their babies, where they undergo Polymerase Chain Reaction (PCR) HIV testing to determine whether the babies are HIV exposed or not [10]. Poor knowledge and awareness of MTCT and PMTCT follow-up services will continue to be a problem unless the gaps are identified and eliminated.

## METHODS

### Study Settings

This study was conducted at two health facilities at Mankweng Township in Limpopo Province. Mankweng is a township located in the Capricorn District Municipality, Limpopo Province, South Africa. Mankweng Gateway clinic and Mankweng Clinic were two of the facilities selected for this study and both clinics provide PMTCT service. HIV infected mothers are referred to the clinics for follow-up services after giving birth in hospitals.

### Research Design

This study applied the qualitative approach and a cross-sectional design to provide a snapshot of the variable included in the study at one particular point in time. In this design, the researcher explored the association between the exposure and the outcome variable [11].

### Study Population and Sampling

The target populations of this study were nurses, lay counsellors, and HIV-positive mothers who were enrolled for PMTCT follow up services in Mankweng Clinics, and who met the eligibility criteria for inclusion in the study. To meet the eligibility criteria: Mothers must be HIV-positive and enrolled into the PMTCT follow up programme for treatment. Nurses and lay counsellors must be trained and have at least two years or more experience in implementing the PMTCT programme. Primary data was gathered once by the authors between the months of June and July 2016. The researcher met with the study respondents and collected qualitative data on knowledge and awareness of MTCT and PMTCT post-natal follow-up services among HIV infected mothers. Voice recording tapes and direct note taking tools were used to collect data.

For the purpose of this study, purposive sampling was used to select participants with a specific purpose in mind. Two health facilities in the Capricorn District were selected. For inclusion in the study, the health facilities met the following criteria: The facilities have HIV and AIDS (Acquired Immune Deficiency Syndrome) counsellors or lay counsellors and nurses responsible for the implementation of the PMTCT programme; and the facilities provide postnatal PMTCT follow-up services. The following procedures were used to select HIV infected mothers: The clinic register checked patients who entered for the day, and patients who reported for PMTCT follow-up services were referred to the researcher`s temporary office in the facility. The researcher was introduced to the patients, and after checking their records, patients who met the criteria were selected. The purpose of the study was explained to each respondent after which they were asked if they were willing to participate in the research. The interview was then conducted after the patients agreed to participate. In the focus group discussions, respondents were approached through the clinics managers. The managers were informed about the study, and appointments were scheduled with health workers (nurse and lay counsellors) for focus group interviews which were conducted at the clinics. The researcher approached the participants in a meeting to discuss with them the research procedures to be followed. Those willing to participate were included in the group. The researcher outlined the purpose of the study and the objectives and then participants were given informed consent forms.

### Sampling Size

The sample size of the study was 26 participants. The participants scheduled for in-depth interviews consisted of fifteen HIV positive mothers enrolled for PMTCT postnatal follow-up services. The participants scheduled for focus group discussions consisted of eleven health workers (nurses and lay counsellors) who worked in the PMTCT programme.

### Data Collection

#### In-depth interviews

Interviews of the selected participants were held at the clinics, interviews lasted approximately 45 minutes for each respondent to allow the researcher to gather more data and to ensure accuracy of information from the respondents. Audio recordings and direct note taking tools were used to collect data. Interviewees were encouraged to talk freely about events, behaviour and beliefs related to PMTCT follow-up service utilisation. The interviews were conducted with fifteen HIV-positive mothers who are enrolled into the PMTCT follow-up services.

### Focus Group Discussion (FGD)

Two focus group discussions were conducted. Each group discussion comprised six and five participants (lay counsellors and nurses) responsible for the implementation of the PMTCT programme. Audio recordings and direct note taking tools were used to collect data. The aim was to get more in-depth information on perceptions, insights, attitudes, experiences, or beliefs from key participants [8, 11]. Data was collected on the participants’ perceptions about loss of follow-up by mothers and their babies to the PMTCT programme and how this affects the programme. The researcher facilitated group discussions to keep the group focused on the topics of interest. The focus group discussions lasted at least 45 minutes each.

### Data Analysis

The researcher applied the thematic data analysis method. In the first stage, the researcher listened to the audio, and read and re-read transcripts in order to become familiar with what the data entails. In stage two, the researcher generated the initial codes by documenting where and how patterns occur. Data was labelled in order to create categories for more efficient analysis. Categorisation helped to overview the data and to enhance its understanding. In stage three, the researcher combined codes into main themes that represent the data. Themes were identified, where data was grouped under different themes in accordance with the categories under which data was collected. In stage four, the researcher looked at how the themes supported the data, and where the analysis seemed incomplete, the researcher went back to find what was missing. In stage five, the researcher defined each theme, the aspects of data that were captured, and the interesting aspects of the themes. The researcher decided on the themes that make meaningful contributions to the study. The researcher conducted “member check” where he went back to the sample at hand to see if their description is an accurate representation.

### Ethical Considerations

The proposal was approved by the University of Limpopo Turfloop Research Ethics committee, reference (TREC/17/2016: PG). The participants were asked to sign the consent form if they agree to participate in the study.

## RESULTS

In this section, themes and categories emerged after the researcher analyzed the data. Nine themes emerged from the analysed data. Relevant quotations from individual participants were used to support the themes. The following themes were identified with HIV exposure mothers and Health personals:

- Knowledge about the stages of MTCT
- Knowledge and awareness of MTCT after birth
- Infection of HIV from an HIV positive mother to her baby
- Knowledge and awareness of the PMTCT programme
- Knowledge about the use of HIV treatment
- Health personnel’s` knowledge of Mother-to-Child Transmission (MTCT)
- Clients` knowledge on HIV transmission from a mother to her baby
- Health personnel`s Knowledge of HIV treatment
- Clients’ Knowledge of HIV treatment

### Demographic Characteristics of the Study Participants

Demographic variables from in-depth interviews with HIV infected mothers show that majority of the participants were between the ages of 26 to 35, and 40% were single, 33% were cohabiting and 27% were married. Of these respondents, 54% achieved secondary and tertiary education 13% had and tertiary education, and 73% were unemployed (see Table **1** below). A total of 8 nurses and 3 lay counsellors from two health facilities participated in the FGDs. The majority of the participants were between the ages of 46 to 50, and 55% were single, 90% were male and 73% were nurses. Of these respondents, 55% obtained a diploma, 27% obtained FET/Collage certificate, 9% had a Matric certificate and only 9% were obtained a degree (see Table **2** below).

### Results From In-Depth Interviews With Mothers

#### Knowledge About the Stages of Mother-to-Child Transmission (MTCT)

Mother-to-Child Transmission (MTCT) occurs when HIV is transmitted from a mother who is infected to her baby through the placenta during pregnancy or through blood contamination during childbirth, or through breast-feeding after birth [4]. In this study, the respondents were aware of the stages where an HIV positive mother can transmit the virus to her baby. However, the majority of the respondents lacked knowledge of how MTCT of HIV occurs in these stages. For example, one respondent reflected on lack of knowledge about MTCT during pregnancy by saying: *“I know that a mother can transmit the virus to her child when she is pregnant, but I don’t know how”*. When asked about how a mother transmits the virus during breastfeeding, another respondent who also lacked knowledge said: "I don't know". One respondent did not believe that a mother can transmit the virus through breastfeeding. This is what she said: *“If I’m not on medication for a long time I can infect the child, not through breastfeeding, breast milk is healthy, they say it protect against diseases, maybe through a cut”.*

The study findings also reflected a level of doubt, as some of the respondents were not sure about how MTCT occurs. One of the participants had this to say: *“I heard, but I don't know what happened. I read somewhere that when there is a cut during operation”*. Another respondent changed her response and did not agree that an HIV positive mother can transmit the virus to her child during pregnancy. This is what she said: *“I doubt that a mother can infect her child during pregnancy, but if the people with HIV have sex without protection infection can occur. I don’t know the process but if there is unprotected sex”.*

#### Knowledge And Awareness of Mother-to-Child Transmission (MTCT) After Birth

This section of the in-depth interview schedule examined the respondents’ knowledge of HIV and MTCT. All the respondents were aware that an HIV-positive mother can transmit HIV to her baby. However, the majority of the respondents lacked knowledge and understanding of how a mother can transmit HIV to her child after birth. For example, one respondent had this to say: *“I don’t know, I don`t know how a mother transmit the virus to her baby after birth”.* Another mother had this to say: *“The mother infects the child through blood from the mother, if there is cut”*. This was supported by another mother who said: *“If I am injured, I can transmit the virus through my blood”.* Other participants at the clinics, however, demonstrated knowledge about MTCT of HIV infection. For example, one respondent had this to say: “It can be transmitted to the baby through breastfeeding if the mother is HIV positive and if the mother is not on treatment”.

#### Infection of HIV from an HIV Positive Mother To Her Baby

The respondents were asked if all babies born to HIV positive mothers always become infected with HIV. The majority of the respondents agreed that all babies born to HIV-positive mothers cannot always become infected. Only one participant reflected lack of knowledge, and this is what she said: *“I don’t know*”. The findings showed that most of the participants were aware that MTCT can be prevented. This was evidenced by the response of the participant, who said: *“It depends on whether the mother was on treatment. It is possible for the child to be negative if the mother was on treatment”*. A similar response was expressed by another participant. This is what she said: "*because if the mother is on treatment, she will protect the baby from infection”.* Another participant responded by saying: *“No, other people don’t get infected, because of HIV treatment”.*

#### Knowledge And Awareness of the PMTCT Programme

The majority of the respondents were aware of interventions that help women to prevent the MTCT of HIV. This was demonstrated by a response from one of the participants, who said: *“I am aware of the PMTCT programme; I heard about it from the radio, it talks about ARV and safe sex”*. Another respondent gave a similar response. This is what she said: *“I know about the PMTCT programme, read about it from books. It helps children to not get infected”*. Other participants did not know what the PMTCT programme is when they were asked to identify programmes that help prevent MTCT. However, the respondents knew that HIV drugs are given to mothers to reduce HIV transmission. One of the participants: *“The one that provide ARV and condom”.* This was supported by another participant who said: *“Provide the treatment before and after birth”*. Other participants were able to identify the programme but could not explain what it entailed. This was demonstrated by a response from one of the participants, who said: *“I know the PMTCT programme; I heard about it from the street but I don’t know what it does”*.

#### Knowledge About the Use of HIV Treatment

All the respondents agreed that the use of anti HIV drugs helps in preventing MTCT of HIV. They were aware of the use of anti HIV drugs as one of the interventions provided to prevent MTCT of HIV. One of the participants had this to say: *“ARV prevents mother-to-child transmission, if not taking the medication is likely that the child will be infected”.* Another respondent gave a similar response, and this is what she said: *“The virus will not affect the child if I am on medication”.* Other respondents provided information from what they observed from other people’s experiences. One of the participants had this to say: *“They work, my sister is HIV positive and got pregnant, by using ARV she end up having a child who is HIV negative”*. A similar response was provided by another participant, and this is what she said: *“My friend took her medication in time and now her child is negative”.*

### Results From Focus Group Discussions With Health Personnels

#### Health Personnel’s` Knowledge of Mother-to-Child Transmission (MTCT)

The participants in the FGDs were asked to discuss the possibilities of HIV transmission from an HIV infected mother to her baby. The participants provided adequate knowledge of MTCT of HIV. HIV-positive mothers can transmit HIV to their babies during pregnancy; the foetus is infected by HIV when it crosses the placenta. This was evidenced by one of the participants, who said: *“If there is depression during pregnancy and rupture of the membrane, transmission can occur through the placenta”*. Another participant had this to say: *“If a mother is pregnant and positive and not using treatment”*

An HIV-positive mother can transmit HIV to her baby during labour, through the mother's cervical secretions or through blood. This was supported by one of the participants, who said: *“During delivery if there is a cut, blood can enter the child”* Similar responses were provided by another participant, who said: *“An HIV-positive mother can transmit HIV to her baby if there is a cut, blood can enter to the child if the mother have a cut”.* The transmission of the HIV virus from an HIV infected mother to her baby can also occur during breastfeeding where the baby is infected through the mother's breast milk (or through blood if there is a cut). This was evidenced by one of the participants, who said: *“Breastfeeding and not taking treatment, if the mother is not on long-life treatment”*. Another participant had this to say: *“During breastfeeding more especially from six month old babies where they are introduced solid food they develop teeth so they bite the mother, that`s where transmission can occur, from the cut”*

#### Clients` Knowledge on HIV Transmission From A Mother To Her Baby

Respondents in the FGDs were asked if they think HIV-positive mothers understand the possibility that they can transmit the HIV virus to their babies. The following responses were given: *“Yes but not 100%”. Another participant had this to say: “Some have this belief that they go somewhere and be cured, that a priest pray for them”.* One of the participants highlighted the issues of disclosure as a challenge for mothers. This is what she said: *“At home you find that clients haven’t disclosed their status, hence they are forced to give their babies medication. So they find it hard to give the child medication in front of family members because they haven’t disclosed their status, so to avoid disclosure of their HIV status they stop treatment”.* By contrast, other respondents believe that their clients have knowledge about the methods of PMTCT. Clients are provided with education in health facilities. This was evidenced by one of the participants, who said: *“They have that knowledge, through education at the facility”*

#### Health Personnel`s Knowledge of HIV Treatment

The findings in the FGDs show that the Health care workers have knowledge about the importance of ARVs intake as a basic strategy that helps mothers from transmitting HIV to their babies. Participants also emphasize the importance of educating their clients to ensure that they prevent MTCT by adhering to treatment. This was evidenced by one of the participants, who said: *“Immediately when the clients come to clinic, and get tested, and then find that she is positive, she is provided with treatment”.* Another participant had this to say *“Intake of ARVs is a lifelong treatment”.*

#### Clients’ Knowledge of HIV Treatment

ART (antiretroviral therapy) is the use of HIV medicines to treat HIV infection. ART is recommended for everyone infected with HIV. ART helps people with HIV to live longer, healthier lives. HIV medicines reduce the amount of HIV in the body. Having less HIV in the body reduces a woman's risk of passing HIV to her child during pregnancy and childbirth [12]. The participants agreed that their clients are taking treatment. This was evidenced by one of the participants, who said: *“In the past clients were not following treatment but now because of education they follow treatment”*

## DISCUSSIONS

PMTCT programmes around the world are hugely affected by the level of awareness and knowledge which consequently increases the prevalence of HIV infections in children [13, 14]. Mothers who lack knowledge and awareness of MTCT may ignore PMTCT follow-up services, and miss out on HIV treatment for both their health and that of their babies. The majority of the participants lacked knowledge of how a mother can transmit HIV to her child after birth. Participants failed to mention breastfeeding as a mode of transmission. This is similar to a study carried out in Lagos, Nigeria which indicates that the majority of women (89.9%) had good knowledge of the mode of HIV transmission; however, specific aspects of PMTCT were poor. Close to half of the study participants (41.7%) were not aware of the association between breast milk and HIV transmission [15].

The findings from this study also reveal that the majority of the respondents lacked knowledge and understanding of how a mother can transmit HIV to her child during pregnancy, labour and breastfeeding. Although basic knowledge of HIV and AIDS seems to be increasing in most communities, there is insufficient knowledge of MTCT among mothers enrolled for PMTCT follow-up services [16, 9]. A study conducted in the Eastern Cape confirmed that knowledge levels about PMTCT were low among mothers, and this affects the uptake of services [8]. In contrast, a study conducted in Uganda revealed that only 12% of the participants lacked knowledge about the possibility of the virus being passed on to an unborn baby, and at least 8% of the participants did not know how the virus can pass from a mother to a child [17]. This is supported by findings from a study conducted in Mpumalanga which revealed that 74.2% of the participants knew that HIV can be transmitted to the child during delivery, while 77.9% knew that it can also be transmitted through breastfeeding [18].

The majority of the participants were aware of interventions that help women to prevent MTCT. Most respondents could not identify the PMTCT programme; they could however identify activities that are provided in the programme. This is supported by a study of 202 women participants who found that at least 94% of the participants have knowledge of HIV and AIDS. However, 48% of the participants were not aware of any means to prevent MTCT [14]. HIV-positive mothers need adequate information in order to make the right decision about their health and that of their babies. Counselling on MTCT and postnatal follow-up practices are provided at health facilities to help mothers prevent HIV transmission after birth, especially during breastfeeding [15]. The findings in the FGDs with health care workers show that the participants have knowledge and understanding of MTCT. A study conducted in Nigeria support the above statement and found that 98.1% of health workers who participated in the study were aware of HIV and MTCT. When asked to mention modes of transmission of HIV, only 20.4% of spontaneously mentioned transmission from an infected mother to child, however, when probed with a closed question, the proportions that were aware of MTCT increased to 90.7% and breastfeeding was most often mentioned as a possible period of MTCT [4].

In-terms of the use of HIV treatment, mothers were aware of the importance of treatment adherence as a prevention measure to avoid MTCT. A study conducted in Mpumalanga reported that only 7.7% of the mothers did not know that AZT and nevirapine NVP reduced the chances of the child contracting HIV [19]. Compliance to medications and clinic follow-ups is very important in preventing HIV transmission, by improving the overall health outcomes of both the mother and their babies [20]. The findings showed that the participants were aware that anti HIV drugs are helpful in preventing MTCT. The respondents could identify the importance of using HIV drugs, and most of them were aware that the use of these drugs can prevent children from being infected by their HIV infected mothers. A study conducted in Mpumalanga found that the consumption of maternal NVP and its administration to the baby were associated with the mother’s knowledge of HIV and PMTCT [19]. A study conducted in Ghana found that most of the respondents had good knowledge on PMTCT and ARVs. Most of the women in the study stated that, the drugs make the virus weak and unable to attack their immune system. The study shows that 88% knew that vertical transmission was preventable whiles only 7.2% did not know that MTCT was preventable [21].

The findings in the FGDs show that health care workers have knowledge about the importance of ARVs intake as a basic strategy that helps mothers from transmitting HIV to their babies. Participants emphasize the importance of educating their clients to ensure that they prevent MTCT by adhering to treatment. Follow-up services help identify HIV infected babies and initialize ARVs medications for infected children [8]. Failure to comply with ARV’s medications and prophylaxis as well as follow-up to clinic visits for medication re-supply and other PMTCT services disrupt the interventions in the course to eliminating paediatric HIV infections [15, 8].

## CONCLUSION

Based on these findings, educational and awareness programmes need to be developed or strengthened on health risks. Mass campaign media should provide information on the importance of PMTCT activities through the use of community radio stations, TV, newsletters, bill boards etc. People need to know more about PMTCT activities and the danger of MTCT. Health personnel need continuous training to provide effective services in terms of information on PMTCT activities.

## Figures and Tables

**Table 1:** Demographic information of mothers.

**Demographic Information**	**Frequency**	**N**
**Age**	Age group of 18-21 Age group of 22-25 Age group 26-30 Age group 31-35 Age group 36-40 Age group 41-46	0 1 5 5 2 2
**Marital Status**	Married Single Cohabitating	4 6 5
**Employment Status**	Employed Unemployed	11 4
**Medical Aids**	None Medical Aids	13 2
**Educational Level**	None Primary Level Secondary Level Tertiary level	0 2 8 5
**Religious Affiliation**	Christianity African Tradition Islam Buddhism	15 0 0 0

**Table 2:** Demographic information of Healthcare workers.

**Demographic Information**	**Frequency**	**N**
**Age**	Age group of 22-25 Age group 26-30 Age group 31-35 Age group 36-40 Age group 46-50 Above the age of 50	1 1 1 2 3 2
**Gender**	Females Male	10 1
**Marital Status**	Married Single	6 5
**Occupation**	Lay Counsellors Nurses	3 8
**Qualification**	Matric FET/Collage Certificate Diploma Degree	1 3 6 1

## LIST OF ABBREVIATIONS

AIDS: = Acquired Immune Deficiency Syndrome
ART: = Antiretroviral therapy
ARV: = Antiretroviral
AZT: = Zidovudine
FGDs: = Focus Group Discussions
HIV: = Human Immunodeficiency Virus
MTCT: = Mother to Child Transmission
NVP: = Nevirapine
PCR: = Polymerase Chain Reaction
PMTCT: = Prevention of Mother to Child Transmission
